# SHARP1 Suppresses Angiogenesis of Endometrial Cancer by Decreasing Hypoxia-Inducible Factor-1α Level

**DOI:** 10.1371/journal.pone.0099907

**Published:** 2014-06-11

**Authors:** Yun Liao, Wen Lu, Qi Che, Tingting Yang, Haifeng Qiu, Huijuan Zhang, Xiaoying He, Jingyun Wang, Meiting Qiu, Yingfen Zou, Wei Gu, Xiaoping Wan

**Affiliations:** 1 Department of Obstetrics and Gynecology, International Peace Maternity & Child Health Hospital Affiliated to Shanghai Jiao Tong University School of Medicine, Shanghai, China; 2 Department of Obstetrics and Gynecology, Shanghai First People’s Hospital Affiliated to Shanghai Jiao Tong University School of Medicine, Shanghai, China; 3 Departments of Pathology and Biobank, International Peace Maternity & Child Health Hospital Affiliated to Shanghai Jiao Tong University School of Medicine, Shanghai, China; Florida International University, United States of America

## Abstract

Recent data support a role for SHARP1, a basic helix-loop-helix transcription repressor, in the regulation of malignant cell behavior in several human cancers. However, the expression and role of SHARP1 during the development of endometrial cancer (EC) remain unclear. Here we show that upregulation of SHARP1 suppressed tumor angiogenesis by decreasing hypoxia-inducible factor-1α (HIF-1α), inhibited cell viability and tumor growth in EC. Immunohistochemical staining showed that the expression of SHARP1 was negatively correlated with tumor stage, histological grade, myometrial invasion, lymph node metastasis, blood vessel permeation in the myometrium and HIF-1α expression. Mechanistic studies showed that SHARP1 interacted with HIF-1α physically, and the protein level of HIF-1α and the mRNA level of its target genes (*VEGFA, ANGPTL4* and *CA9*) were decreased by SHARP1 under hypoxia. Upregulation of SHARP1 in EC impeded hypoxia-induced angiogenesis by reducing VEGF secretion. Immunohistochemical analysis verified a correlation between decreased SHARP1 expression and increased microvessel density in EC tissues. Additionally, SHARP1 inhibited cell viability in EC cell lines. Overexpression of SHARP1 in vivo inhibited tumor growth and angiogenesis, and decreased HIF-1α expression. In this study, we established SHARP1 as a novel tumor suppressor of EC and shed light on the mechanisms by how SHARP1 inhibited EC progression. Therefore, SHARP1 may be a valuable prognostic biomarker for EC progression and shows promise as a new potential target for antiangiogenic therapeutics in human EC.

## Introduction

Endometrial cancer (EC) is a major cause of cancer-related morbidity and mortality among women. It is the fourth most common malignancy among women in the United States, with an estimated 49,500 new cases and 8200 deaths in 2013 [1]. Although early-stage EC is often successfully treated with surgical intervention, treatment of advanced EC can be difficult and its prognosis is poor [2], [3], particularly in the context of metastatic or recurrent disease, with a median survival of only 7–12 months [4]. Current treatment options, such as hysterectomy, hormonal therapy, and combinations of radiation and chemotherapy, are effective for early-stage EC; however, effective target therapies for patients with metastatic or recurrent EC are unavailable [5], because the etiology and biological mechanisms of this heterogeneous disease are not fully understood. Therefore, further elucidation of the molecular mechanisms underlying EC progression is urgent.

Angiogenesis, the formation of new blood vessels from preexisting ones, has a major role in tumor growth, progression, and metastasis [6], [7]. Initially, angiogenic activity is absent in early neoplasms. However, as tumors grow beyond the existing supply rate, oxygen becomes exhausted within the core. The resulting hypoxic condition (PO_2_<10 mmHg [∼1.5% O_2_]) prevents degradation of hypoxia-inducible factor-1α (HIF-1α) through VHL tumor suppressor, allowing trans-activation of pro-angiogenic factors, among which the most notable is vascular endothelial growth factor (VEGF) [8]–[11]. Recent research has demonstrated that the HIF-1α/VEGF signaling pathway is involved in endothelial cell proliferation, differentiation, migration, and vascular permeability [12]. Moreover, inhibition of neovascularization by suppression of the HIF-1α/VEGF signaling pathway may delay tumor progression and perhaps even starve tumor cells to death [13]–[15]. Like other solid tumors, the growth and metastasis of EC cells depend on angiogenesis [16]. The mechanism underlying angiogenesis regulation has been extensively described in other types of cancer but only scarcely studied in EC [15].

SHARP1 (also known as bHLHE41 or DEC2), a member of the transcriptional repressor subfamily of basic helix-loop-helix (bHLH) transcription factors [17], [18], is expressed in various embryonic and adult tissues [19], [20]. Extensive evidence suggests it is a key regulator of tumor progression in oral and breast cancer, where its effects on cancer cell apoptosis, proliferation, and metastasis have been widely investigated [21]–[24]. In addition, SHARP1 negatively regulates VEGF expression [25], and a recent study shows that SHARP1 suppresses breast cancer metastasis [23]. However, it remains unknown whether and, if so, how SHARP1 contributes to the development and progression of EC, especially with respect to angiogenesis, in which the HIF-1α/VEGF pathway is involved.

In the present study, we demonstrated that decreased expression of SHARP1 was significantly correlated with poor clinical outcome in EC. Moreover, for the first time, we showed that SHARP1 has a suppressive role during EC progression; in particular, SHARP1 inhibited angiogenesis in a HIF-1α-dependent manner and might be a valuable marker for antiangiogenic therapy selection.

## Materials and Methods

### Ethics Statement

This study was approved by the Human Investigation Ethical Committee of the International Peace Maternity & Child Hospital affiliated to Shanghai Jiao Tong University. The samples of EC and normal endometrial tissues were collected after receiving written informed consent from the patients. The animal research was carried out in strict accordance with the recommendations in the Guideline for the Care and Use of Laboratory Animals of China. The procedures were approved by the Department of Laboratory Animal Science Shanghai Jiao Tong University School of Medicine. All efforts were made to minimize animal suffering.

### Patients and Samples

Paraffin-embedded tissues samples were obtained from 110 patients with endometrial cancer and 52 patients with normal endometrium who underwent surgical resection to treat other diseases such as myoma or adenomyosis at the International Peace Maternity & Child Health Hospital affiliated to Shanghai Jiao Tong University from 2009 to 2013. The average age of the patients with EC was 56.9±8.8 years (mean ± SD; median, 57 years; range, 33–78 years). The average age of the patients with normal endometrium was 53.9±8.6 years (mean ± SD; median, 53 years; range, 34–70 years). There was no significant difference regarding patient age when EC and control samples were compared (*P* = 0.054). In control group, all samples were hysterectomy specimen, among which 16 cases were from postmenopausal women, 17 cases were from perimenopausal women and the others were from premenopausal women. The stages (I–IV) and histological grades (G1–G3) of these tumors were established according to the criteria of the International Federation of Gynecology and Obstetrics (FIGO) surgical staging system (2009) [26]. None of the patients had undergone hormone therapy, radiotherapy, or chemotherapy before surgery.

### Immunohistochemistry (IHC) and Assessments

Tissue sections (4 µm thick) were prepared from paraffin-embedded tissue specimens. The sections were dewaxed with xylene followed by rehydration in graded alcohol. Antigen retrieval was carried out in ethylenediaminetetraacetic acid (EDTA; pH 9.0) for 20 min by heating in a microwave oven. Then endogenous peroxidase activity was blocked by incubating the sections with 3% hydrogen peroxide for 10 min, and nonspecific staining was blocked by incubation with 10% normal goat serum for 30 min. Sections were incubated with rabbit polyclonal antibody against SHARP1 (a dilution ratio of 1∶100 to 10 µg/ml; Novus, Littleton, CO), mouse monoclonal antibody against HIF-1α (a dilution ratio of 1∶50 to 5 µg/ml; BD Biosciences, Bedford, MA), rabbit monoclonal antibody against CD34 (a dilution ratio of 1∶100 to 5 µg/ml; Abcam, Cambridge, UK), and rabbit polyclonal antibody against Ki67 (a dilution ratio of 1∶100 to 5 µg/ml; Epitomics, Burlingame, CA) in a humidified chamber at 4°C overnight and then were incubated with biotinylated secondary antibody (a dilution ratio of 1∶1000 to 1 µg/ml; Beyotime, Nanjing, China) for 30 min, followed by streptavidin peroxidase for 15 min. Phosphate-buffered solution (PBS) was used to dilute antibodies. Peroxidase activity was detected by applying 3,3′-diaminobenzidine tetrachloride. Each incubation step was performed at 37°C and was followed by three sequential washes with PBS for 5 min each. Finally, sections were dehydrated in alcohol and cleared in xylene.

Scoring was performed by two independent pathologists who were blinded to the clinical and pathological data. SHARP1 staining was evaluated by considering both the number of cells with positive nuclear and cytoplasmic staining and the intensity of the staining in each positive nucleus and cytoplasm. The number of positive cells was graded as follows: 0 (<5%); 1 (5–25%); 2 (26–50%); 3 (51–75%); 4 (>75%). The intensity was graded as follows: 0 (negative); 1 (weak); 2 (moderate); 3 (strong). A final score was calculated by multiplying the two scores above. Scores of 0–2 were defined as ‘‘negative expression’’; scores of 3–8 as ‘‘weakly positive expression’’, and scores of 9–12 as ‘‘strongly positive expression’’ [27]. For HIF-1α staining, only the percentage of dark, homogenously stained nuclei was assessed, and cytoplasmic staining was ignored. It was considered positive when >1% of nuclei were stained [28]. The microvessel density (MVD) was assessed by IHC staining for CD34, which highlights the tumor/tissue vascularity. The microvessels were counted in three different fields per section as follows: slides were first scanned under low power (×100) to determine three ‘‘hotspots’’ or areas with the maximum number of microvessels, then the positive-stained blood vessels in the selected areas were analyzed at ×400 magnification [29].

### Cell Culture, Hypoxic Conditions, and Reagents

Human EC cell lines (Ishikawa and RL95-2) were obtained from American Type Culture Collection (ATCC, Manassas, VA). Human umbilical vascular endothelial cells (HUVECs) were obtained from Key-GEN Biotech (Nanjing, China). Ishikawa and RL95-2 cells were grown in DMEM/F12 (Gibco, Auckland, NZ) supplemented with 10% fetal bovine serum (Gibco, Carlsbad, CA). HUVECs were cultured in RPMI1640 (Gibco) supplemented with 10% fetal bovine serum. Cells were cultured in a 5% CO_2_ humidified incubator at 37°C. For hypoxia experiments, cells were grown in an in vitro hypoxic (<0.1% O_2_) container system (BD Diagnostics, Sparks, MD). Conditioned medium (CM), protein and RNA were collected immediately when cells were removed from hypoxic container.

### Transient and Stable Transfections for SHARP1-overexpression

The SHARP1 expression plasmid pEZ-M02-SHARP1 and empty plasmid pReceiver-M02-NEG were purchased from GeneCopoeia (Guangzhou, China). Transient transfection was performed in 70% confluent Ishikawa cells using Lipofectamine 2000 reagent (Invitrogen, Carlsbad, CA) according to the manufacturer’s protocol. For stable expression in Ishikawa cells, the human SHARP1 gene was cloned into lentiviral vectors with Ubi-MCS-3FLAG-SV40-EGFP using Gateway technology (Invitrogen, Carlsbad, CA) according to the operation manual (http://products.invitrogen.com/ivgn/product/12538120).

### small interfering RNA (siRNA)

siRNAs were synthesized by GenePharma Biotech (Shanghai, China). The sequences are presented in Table 1. The siRNA was transfected into cells using Lipofectamine 2000 reagent as described above.

**Table 1:** siRNA sequences.

siRNA	Sequence
Negative control	UUCUCCGAACGUGUCACGU
SHARP1	GCUUUAACCGCCUUAACCG
HIF-1α	GAAUUCUCAACCACAGUGC

### RNA Isolation and Quantitative Real-time PCR (qPCR)

Total RNA was extracted from cultured cells using Trizol (Invitrogen, Carlsbad, CA). First-strand cDNA was reverse-transcribed from 1 µg of total RNA using the Prime Script RT reagent kit (TaKaRa, Dalian, China), and the cDNA was analyzed by qPCR using SYBR Premix Ex Taq (TaKaRa). For qPCR experiments, values on the *y* axis represent 2^(−ΔΔCt)^, where ΔCt is the difference between the Ct for the gene of interest and that of the gene encoding β-actin and ΔΔCt is the difference between the ΔCt of the experimental group and control group or first lane [30]. Primer sets are shown in Table 2. Data were obtained in triplicate from three independent experiments.

**Table 2:** Primer sequences for qPCR.

Gene	Sequence
*SHARP1*	Forward 5′-GCATGAAACGAGACGACACC-3′
	Reverse 5′-CGCTCCCCATTCTGTAAAGC-3′
*VEGFA*	Forward 5′-CCTTGCCTTGCTGCTCTACCTC-3′
	Reverse 5′-TTCTGCCCTCCTCCTTCTGC-3′
*CA9*	Forward 5′-TGGCTGCTGGTGACATCCTA-3′
	Reverse 5′-TTGGTTCCCCTTCTGTGCTG-3′
*ANGPTL4*	Forward 5′-TCCGCAGGGACAAGAACTG-3′
	Reverse 5′-GCCGTTGAGGTTGGAATGG-3′
*HIF-1α*	Forward 5′-TCATCCAAGAAGCCCTAACG-3′
	Reverse 5′-TCGCTTTCTCTGAGCATTCTG-3′
*β-actin*	Forward 5′-CTGGGACGACATGGAGAAAA-3′
	Reverse 5′-AAGGAAGGCTGGAAGAGTGC-3′

### Western Blotting

Cells were lysed in RIPA lysis buffer (Beyotime, Nanjing, China) with protease inhibitor phenylmethanesulfonyl fluoride (PMSF; Beyotime). Protein concentration was determined by a BCA Protein Assay kit (Beyotime). Equal amounts of protein were loaded into each lane of a SDS-PAGE gel for protein separation and transferred to polyvinylidene fluoride (PVDF) membranes (Millipore, Billerica, MA). Membranes were blocked and then incubated with rabbit polyclonal antibody against SHARP1 (a dilution ratio of 1∶500 to 2 µg/ml; Novus),mouse monoclonal antibody against HIF-1α (a dilution ratio of 1∶200 to 5 µg/ml; Abcam), and rabbit polyclonal antibody against β-actin (a dilution ratio of 1∶5000 to 0.2 µg/ml; Epitomics) individually at 4°C overnight. Then peroxidase-linked secondary anti-rabbit and anti-mouse antibodies (a dilution ratio of 1∶5000 to 0.2 µg/ml; Cell Signaling Technology, Beverly, MA) were used to detect the bound primary antibodies.

### Co-Immunoprecipitation (Co-IP) Assay

To study endogenous SHARP1/HIF-1α binding, Ishikawa cells were incubated under hypoxia for 24 h before harvesting. Then two confluent 10-cm dishes of cells were lysed as described above. Prior to immunoprecipitation, total cell lysates were incubated with 1 µg mouse IgG and 20 µl of a protein A+G-agarose bead slurry (Beyotime) at 4°C for 2 h to eliminate nonspecific binding. Centrifugation was then carried out and the supernatant was collected and incubated with mouse anti-human HIF-1α (final concentration: 10 µg/ml; Abcam) or control mouse IgG (final concentration: 10 µg/ml; Beyotime) overnight. The next day, 40 µl of a protein A+G-agarose bead slurry was added to the reaction mixtures and incubated for 4 h at 4°C with rotation. Co-immunoprecipitated proteins were analyzed by western blotting as described above.

### Enzyme-Linked Immunosorbent Assay (ELISA)

Quantikine human VEGF kit was purchased from R&D systems (Minneapolis, MN). Cells were seeded in 12-well plates. At 24 h post-transfection, the medium was replaced by DMEM/F12, and cells were cultured under hypoxia or normoxia for another 24 h. Then the supernatants were collected, and extracellular VEGF in the medium was quantified according to the manufacturer’s instructions.

### Endothelial Cell Tube Formation Assay

A 96-well plate was coated with 100 µl Matrigel (BD Biosciences, San Diego, CA) per well and kept at 37°C for 30 min. Then, 2×10^4^ HUVECs were suspended in 1 ml diluted conditioned medium (CM; 1∶10) and applied to the pre-coated 96-well plate at a density of 2000 cells/well. CM was collected from the supernatant of Ishikawa cells cultured under hypoxia or normoxia for 24 h. After incubation at 37°C for 14 h, the total length of the capillary-like tubes that had formed were counted and averaged in randomly selected three microscopic fields. Morphologic changes were observed under a microscope, and cells were photographed at 100× magnification.

### Nude Mouse Tumor Xenograft Assay

Nineteen 6-week-old female nude BALB/c mice were obtained from Shanghai Life Science Institute (Slac Laboratory Animal Co., Ltd, China). To establish a nude mouse model bearing EC, Ishikawa cells transfected with lentiviral vector carrying the human SHARP1 gene (Lenti-SHARP1) or with empty vector alone (Lenti-Control) were used. All mice were randomly divided into two groups of five mice (the other nine mice were used for the Matrigel plug assay; see below). The cells were injected subcutaneously into the flank of each mouse (1×10^7^ cells per injection). Subcutaneous tumor formation was monitored in all nude mice every 7 days from 14 days after the injection. The short and long diameters of the tumors were measured using a caliper and tumor volumes (cm^3^) were calculated by using the following standard formula: tumor volume (cm^3^)  =  (the longest diameter) × (the shortest diameter)^2^×0.5. Mice were sacrificed 6 weeks post-injection, after which tumors were carefully removed, and their weights and volumes were measured prior to further histological evaluation.

### Mouse Matrigel Plug Assay

Nine nude BALB/c mice were randomly divided into three groups of three mice. Growth factor-reduced Matrigel (BD Biosciences) (300 µl) was mixed with Ishikawa cells transfected with empty plasmid under normoxia or hypoxia or SHARP1 full-length plasmid under hypoxia, or mixed with corresponding CMs (100 µl) from the very same cells mentioned above. Cells or corresponding CMs were collected at the same time after cultured under indicated conditions for 24 h. The mixtures were injected subcutaneously into the flank of each mouse (right side, mixture with cells; left side, mixture with corresponding CM). The mice were sacrificed and plugs were removed 14 days after inoculation and photographed. Matrigel plugs were stained with hematoxylin and eosin (H&E) for histological examination and analyzed by IHC as described above using antibodies against mouse CD34 (1∶100, Abcam).

### Trans-well Migration Assays

For HUVEC trans-well migration assays, 1×10^4^ cells suspended in 200 µl of RPMI1640 were seeded on the top chamber and conditioned medium (CM) from different sources as described in the Materials and Methods was added to the bottom chamber, where Ishikawa cells were cultured under normoxia or hypoxia for 24 h before CM was collected. Each sample was plated in triplicate. After 24 h of incubation, cells in the upper side of the top chamber were removed with a cotton swab. The migrating cells (on the lower side of the filter) were fixed in 4% paraformaldehyde and stained with 0.5% crystal violet, and then the number of migrating cells was counted. A total of six fields were counted for each trans-well filter. Each field was counted under a microscope and photographed at 200× magnification.

### Methyl Thiazolyl Tetrazolium (MTT) Assays

Transfected cells were seeded into 96-well plates at 5000 cells/well and were cultured for 1 to 4 d. During the final 4 h of culture, the methyl thiazolyl tetrazolium (MTT; 5 mg/ml) reagent (Sigma, St. Louis, MO) was added to the culture medium. Absorbance values were measured at 490 nm with a microplate reader (model 680; Bio-Rad, Hercules, CA). For HUVEC viability detection, 2×10^4^ HUVECs were suspended with 1 ml diluted CM (dilution ratio 1∶10) and seeded in 96-well plates at 2000 cells/well. After 24 h of incubation, HUVEC viability was detected by the MTT assay as described above. In each experiment and under each condition, cell viability was assessed in triplicate, and experiments were repeated three times.

### Statistical Analysis

All statistical analyses were done using SPSS software version 17.0 (Chicago, IL). Values represent the mean ± SD. Data were analyzed by unpaired Student’s *t*-test or by one-way analysis of variance (ANOVA) for multiple comparisons. The χ^2^ test for tables was used to compare the categorical data. The spearman’s correlation coefficient test was used for correlation detection. A *P*-value of <0.05 was considered statistically significant.

## Results

### SHARP1 is Downregulated in EC and is Correlated with Clinicopathological Characteristics

**Figure 1:**
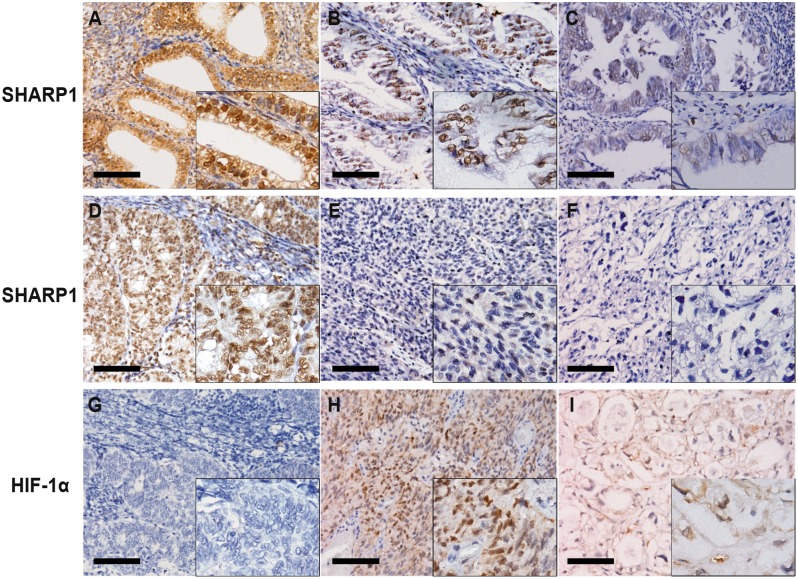
SHARP1 and HIF-1α expression in human endometrium tissue specimens. Representative microphotographs of SHARP1 and HIF-1α staining in endometrium tissues (original magnification: 400× for the inserts, 200× for all others; scale bar, 100 µm). Panels A–C show immunohistochemical staining for SHARP1 in normal endometrium (A), endometrioid endometrial cancer (EEC) (B), and papillary serous EC (C). Panels D–F show immunohistochemical staining for SHARP1 in histological grade 2 (G2) EEC (D), G3 EEC (E), and clear cell EC (F). Panels G–I show immunohistochemical staining for HIF-1α in G2 EEC (G), G3 EEC (H), and clear cell EC (I) in the corresponding same areas of same cases to SHARP1 staining.

To determine the effect of SHARP1 expression on EC development and progression, we chose 52 cases of normal endometrium tissue, 97 cases of endometrioid endometrial cancer (EEC) tissue and 13 cases of papillary serous EC or clear cell EC tissue as representatives for type I and type II EC, respectively. Type I EC, occurring in about 85% of patients, often displays ER positive. Tumors with this type tend to be well differentiated, of low grade and good prognosis. In contrast, type II, consisting mostly of serous and clear cell carcinoma, typically arises in atrophic endometrium via a mechanism unrelated to estrogen exposure. This type is usually ER negative, and poorly differentiated, of high grade and poor prognosis. Although type II ECs account for approximately 15% of cases, they are responsible for about 50% of all relapses [31]. Immunohistochemical staining revealed that SHARP1 was strongly expressed in the cytoplasm and nucleus of most cells in normal endometrium but showed significantly reduced expression in EC (*P* = 0.00046, Table 3; Figure 1A–C).

**Table 3:** SHARP1 expression levels in different tissue specimens.

	SHARP1 expression				
Tissue type	Strong (%)	Weak (%)	Negative (%)	?^2^	*P*
**Normal endometrium**	32 (61.5%)	20 (38.5%)	0 (0%)	15.389	0.00046
**(** ***n*** ** = 52)**					
**Endometrial cancer**	35 (31.8%)	64 (58.2%)	11 (10.0%)		
**(** ***n*** ** = 110)**					

The clinicopathological characteristics of the 110 EC patients were summarized in Table 4. Compared with EEC, SHARP1 expression was significantly decreased or lost in papillary serous EC or clear cell EC (*P* = 0.017), which accounts for the most aggressive types of EC. Moreover, SHARP1 expression decreased markedly in late-stage (stage III or IV) or high-grade (G3) EC as compared with early-stage (stage I or II) or low-grade (G1, G2) EC (*P* = 0.017 and *P* = 0.001, respectively). Additionally, we also found strong inverse correlations between SHARP1 expression and myometrial invasion (*P* = 0.002), lymph node metastasis (*P* = 0.005), and blood vessel permeation in the myometrium (*P* = 0.021). We did not, however, find any significant correlations between SHARP1 and other clinicopathologic variables, including age and expression of estrogen receptor, progesterone receptor, and p53.

**Table 4:** Correlation of SHARP1 expression with clinicopathological characteristics in endometrial cancer.

	Total	SHARP1 expression				
Characteristic	(*n* = 110)	Strong (%)	Weak (%)	Negative (%)	?^2^	*P*
		*n* = 35	*n* = 64	*n* = 11		
**Age**						
** ≤55**	47	18 (38.3%)	25 (53.2%)	4 (8.5%)	1.616	0.446
** >55**	63	17 (27.0%)	39 (61.9%)	7 (11.1%)		
**Pathology**						
** Endometrioid**	97	35 (36.1%)	54 (55.7%)	8 (8.2%)	8.102	0.017
** papillary serous**	13	0 (0%)	10 (76.9%)	3 (23.1%)		
** or clear cell**						
**Grade of EEC**						
** G1**	63	25 (39.7%)	34 (54.0%)	4 (6.3%)	19.840	0.001
** G2**	29	10 (34.5%)	18 (62.1%)	1 (3.4%)		
** G3**	5	0 (0%)	2 (40.0%)	3 (60.0%)		
**Myometrial**						
**invasion**						
** ≤1/2**	78	31 (39.7%)	43 (55.1%)	4 (5.1%)	12.087	0.002
** >1/2**	32	4 (12.5%)	21 (65.6%)	7 (21.9%)		
**Lymph node**						
**metastasis**						
** Negative**	103	35 (34.0%)	60 (58.3%)	8 (7.8%)	10.451	0.005
** Positive**	7	0 (0%)	4 (57.1%)	3 (42.9%)		
**Blood vessel** **permeation in the** **myometrium**						
** Negative**	90	32 (35.6%)	52 (57.8%)	6 (6.7%)	7.687	0.021
** Positive**	20	3 (15.0%)	12 (60.0%)	5 (25.0%)		
**FIGO stage**						
** I or II**	100	35 (35.0%)	57 (57.0%)	8 (8.0%)	8.164	0.017
** III or IV**	10	0 (0%)	7 (70.0%)	3 (30.0%)		
**ER expression**						
** Negative**	19	3 (15.8%)	12 (63.1%)	4 (21.1%)	4.758	0.093
** Positive**	91	32 (35.2%)	52 (57.1%)	7 (7.7%)		
**PR expression**						
** Negative**	18	3 (16.7%)	12 (66.7%)	3 (16.7%)	2.776	0.250
** Positive**	92	32 (34.8%)	52 (56.5%)	8 (8.7%)		
**p53 expression**						
** Negative**	78	27 (34.6%)	46 (59.0%)	5 (6.4%)	4.143	0.126
** Positive**	32	8 (25%)	18 (56.3%)	6 (18.7%)		

ER, estrogen receptor; PR, progesterone receptor.

### Expression of SHARP1 is Inversely Correlated with HIF-1α in EC

To determine whether the reduced SHARP1 expression correlates with HIF-1α expression in tumor tissues, immunohistochemical staining of HIF-1α was performed in 104 of the 110 EC tissue specimens (the rest six specimens were missing or damaged). Among them, 31 were strongly positive for SHARP1 (29.8%), and 77 of 104 specimens were positive for HIF-1α (74.0%). Only 16 cases were strongly positive for SHARP1 and positive for HIF-1α (15.4%). Our clinical data provide the first evidence that there is a strong inverse correlation between SHARP1 and HIF-1α expression in EC (*P* = 0.003, Table 5; Figure 1D–I).

**Table 5:** Correlation between SHARP1 and HIF-1α expression levels in EC.

	HIF-1α staining			
SHARP1 staining	Negative	Positive	Total	Positive rate^a^
**Negative**	1 (1.0%)	8 (7.7%)	9 (8.7%)	88.9%
**Weak**	11 (10.6%)	53 (51.0%)	64 (61.5%)	82.8%
**Strong**	15 (14.4%)	16 (15.4%)	31 (29.8%)	51.6%
**Total**	27 (26.0%)	77 (74.0%)	104 (100%)	74.0%

a There was a significant difference among HIF-1α positive rate of SHARP1 negative staining EC, SHARP1 weak staining EC and SHARP1 strong staining EC (*P* = 0.003).

### SHARP1 Attenuates the Expression of HIF-1α Protein and its Downstream Genes in EC Cell Lines

**Figure 2:**
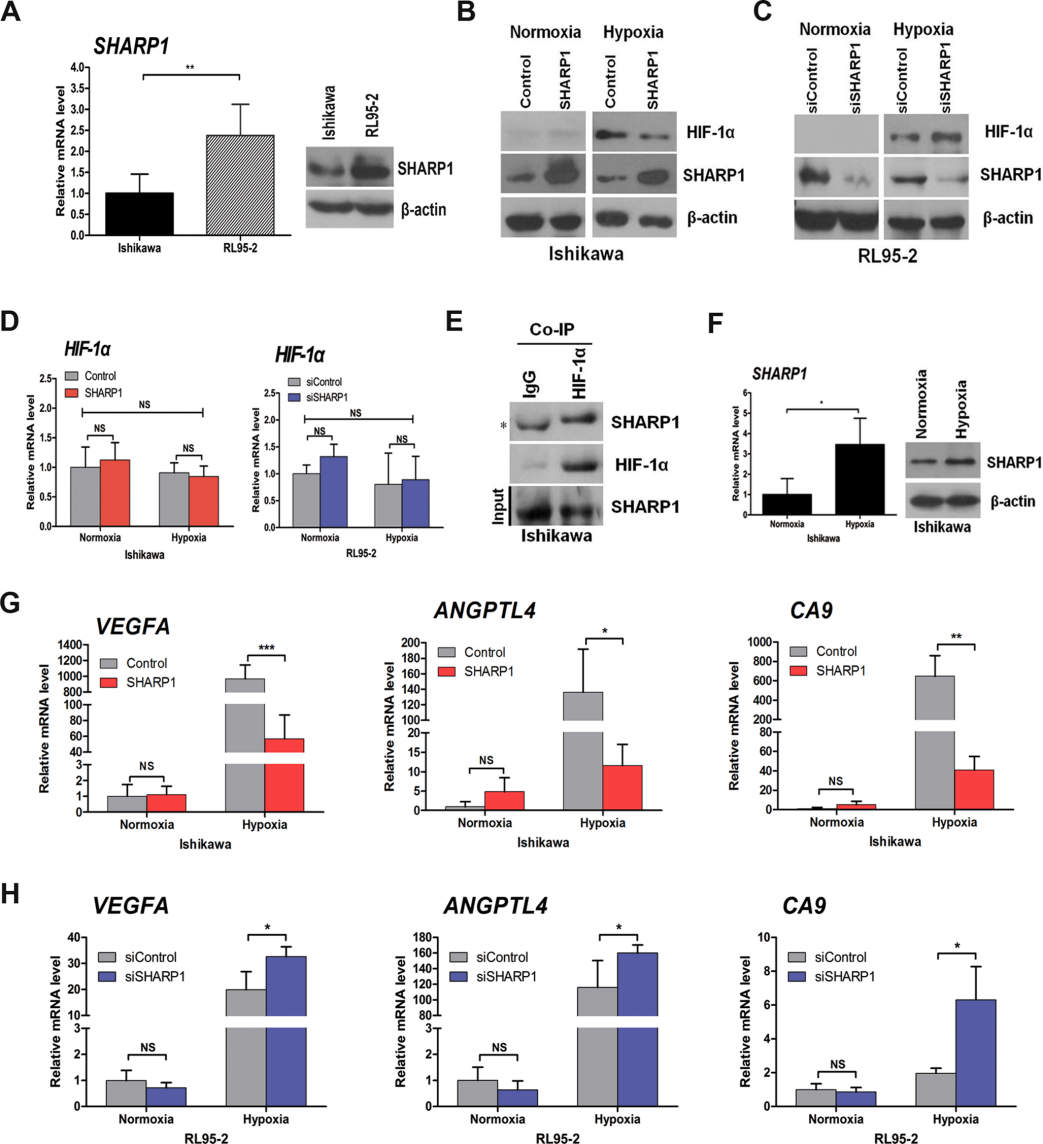
The impact of SHARP1 on HIF-1α protein and its downstream mRNAs. (A) Expression of SHARP1 in Ishikawa and RL95-2 cells as determined by qPCR (left) and western blotting (right). (B) Western blot analysis of control and SHARP1-overexpressing Ishikawa cells cultured at the indicated oxygen levels for 24 h. (C) Western blot analysis of the effects of SHARP1 depletion in RL95-2 cells after 24 h at the indicated oxygen levels. (D) qPCR analysis of *HIF-1α* mRNA in control and SHARP1-overexpressing Ishikawa cells (left), and in RL95-2 cells that had been transfected with control or SHARP1 siRNAs (right) incubated at the indicated oxygen levels for 24 h. (E) Co-immunoprecipitation (Co-IP) of endogenous HIF-1α with endogenous SHARP1 from extracts of Ishikawa cells. The asterisk indicates a background band resulting from the cross-reaction of immunoglobulins (IgGs). (F) qPCR (left) and western blotting (right) analysis of *SHARP1* in Ishikawa cells incubated at the indicated oxygen levels for 24 h. (G) qPCR analysis of *VEGFA*, *ANGPTL4*, and *CA9* in control and SHARP1-overexpressing Ishikawa cells incubated at the indicated oxygen levels for 24 h. (H) qPCR analyses of *VEGFA*, *ANGPTL4*, and *CA9* in RL95-2 cells that had been transfected with control or SHARP1 siRNAs and incubated at the indicated oxygen levels for 24 h. For transient transfections, plasmid (1.6 µg/ml) and siRNA (40 pmol/ml) were used. Data represent the mean ± SD from one representative experiment of three independent experiments, each performed in triplicate (*P<0.05, **P<0.01, ***P<0.001; NS, not significant). For all qPCR analyses, expression levels were compared to β-actin, and data were normalized to the expression shown in the first column.

On the basis of our clinical findings, we proposed a potential relationship between SHARP1 and HIF-1α. Using qPCR and western blotting, we found that the *SHARP1* level was elevated twofold in the EC cell line RL95-2 as compared with the Ishikawa cell line, both of which originated from adenocarcinoma of the endometrium (Figure 2A). To further investigate SHARP1 function and its correlation with HIF-1α, we upregulated SHARP1 using SHARP1 expression plasmids in Ishikawa cells and knocked down SHARP1 using siRNA in RL95-2 cells. Transfection efficiency was confirmed at 48 h and 72 h post-transfection for mRNA and protein levels, respectively (Figure S1).

SHARP1 overexpression significantly decreased HIF-1α protein level in Ishikawa cells under hypoxia (Figure 2B). We then examined whether endogenous SHARP1 was a relevant inhibitor of HIF-1α protein levels. SHARP1 depletion using siRNA increased HIF-1α protein level in RL95-2 cells under hypoxia (Figure 2C). However, HIF-1α protein was hardly detected in both cell lines cultured under normoxia. Meanwhile, no significant change in the *HIF-1α* mRNA level was observed in Ishikawa cells overexpressed SHARP1 or RL95-2 cells with depletion of SHARP1 (Figure 2D). We also detected a physical association between HIF-1α and SHARP1 at endogenous levels in Ishikawa cells under hypoxia using a co-immunoprecipitation assay (Figure 2E). Intriguingly, *SHARP1* expression was elevated in Ishikawa cells after they were cultured under hypoxia for 24 h (Figure 2F). Moreover, HIF-1α knockdown using siRNA decreased SHARP1 expression under hypoxia (Figure S2A).

To test whether SHARP1 functionally affects HIF-1α activity, qPCR was used to detect differences in the levels of selected HIF-1α target gene transcripts. We evaluated the genes that encode vascular endothelial growth factor A (*VEGFA*, an isoform of VEGF that is crucial for tumor angiogenesis and plays a key role in endothelial cell proliferation, survival, and permeability [7], [10], [12], [16], [32]), angiopoietin-like 4 (*ANGPTL4*), and carbonic anhydrase 9 (*CA9*). Notably, these genes were transcriptionally upregulated under hypoxia in Ishikawa cells, whereas SHARP1 expression dampened these inductions (Figure 2G). Conversely, SHARP1 knockdown stimulated the transcription of these genes in RL95-2 cells under hypoxia (Figure 2H). The expression of these genes was, however, not significantly affected by SHARP1 upregulation or downregulation under normoxia. Additionally, downregulation of HIF-1α using siRNA attenuated the mRNA expression of *VEGFA*, *ANGPTL4* and *CA9* in Ishikawa cells under hypoxia (Figure S2B).

### SHARP1 Inhibits Angiogenesis

**Figure 3:**
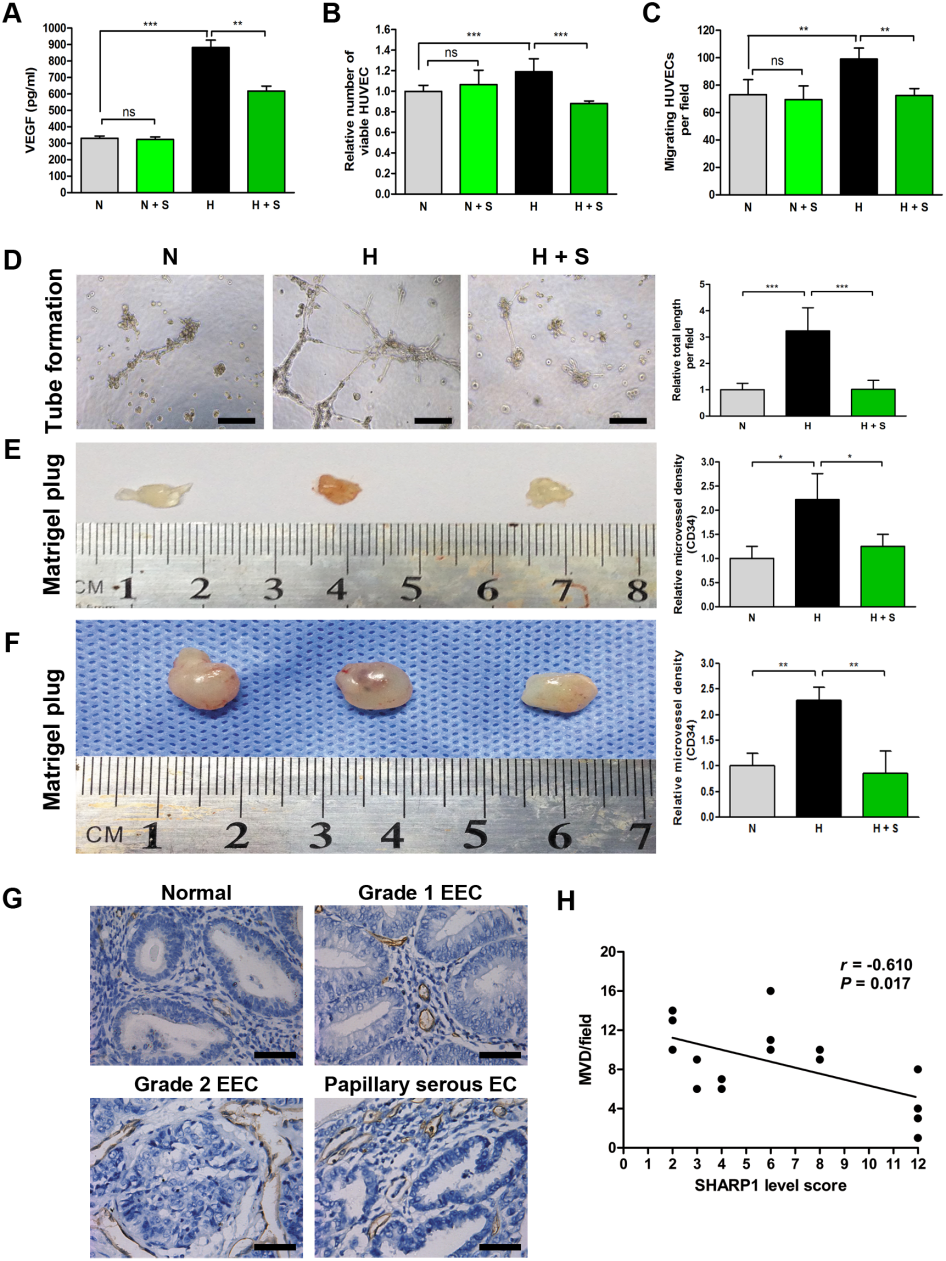
SHARP1 inhibits angiogenesis. (A) The concentration of VEGF in the CMs collected from Ishikawa cells transfected with empty plasmid (1.6 µg/ml) under normoxia (N) and hypoxia (H), or Ishikawa cells transfected with SHARP1 full-length plasmid (1.6 µg/ml) under normoxia (N + S) and hypoxia (H + S) were determined by ELISA. Viability (B), trans-well migration (C) and tube formation (D) of HUVECs, and Matrigel plug assays (E) using the CM described above. (B) MTT and (C) migration assays were performed 24 h after incubation. (D) 96-well dishes were coated with Matrigel, and HUVEC tube formation assays were performed 14 h after incubation. Left, representative photographs are shown at 100× magnification (scale bar, 200 µm). Right, the relative total tube length formed under the indicated conditions. Matrigel plug assays were performed using CMs (E) or corresponding Ishikawa cells described above (F). CMs (100 µl) or cells (1×10^6^ per injection) mixed with Matrigel (300 µl) were inoculated into the flank of a nude mouse, and the Matrigel plug was excised 14 days later. Left, representative Matrigel plugs. Right, relative MVD as determined by immunohistochemical staining for CD34. Data represent the mean ± SD from one representative experiment of three independent experiments, each performed in triplicate (*P<0.05, **P<0.01, ***P<0.001). (G) Representative photographs of CD34 staining in various endometrium tissue specimens (magnification, 400×; scale bar, 50 µm). (H) Spearman’s correlation test was used to analyze the correlation of SHARP1 immunostaining levels and MVD (based on CD34 staining) from 16 EC specimens using SPSS software.

HIF-1α is a master regulator of tumor angiogenesis [33], and SHARP1 inhibited *VEGFA* mRNA expression in our study (Figure 2G, left panel). Moreover, we performed ELISA to detect VEGF secretion in conditioned medium (CM) of Ishikawa cells. SHARP1 overexpression partially decreased the VEGF secretion induced by hypoxia in Ishikawa cells (Figure 3A). Thus, we considered whether the impingement of SHARP1 on HIF-1α/VEGF expression affects hypoxia-triggered angiogenesis in EC. We used several approaches to investigate whether and how SHARP1 could regulate the behavior of endothelial cells. CM was collected from Ishikawa cells transfected with empty plasmid or SHARP1 full-length plasmid under normoxia or hypoxia for 24 h. Notably, the viability and migration ability of HUVECs were stimulated by CM from hypoxic Ishikawa cells, whereas CM from hypoxic Ishikawa cells that overexpressed SHARP1 inhibited these effects (Figure 3B and C). As no obvious effects of SHARP1 on VEGF secretion, HUVEC viability and migration were observed under normoxia, we used CM from Ishikawa cells transfected with SHARP1 or empty plasmids under hypoxia for following experiments, and CM from Ishikawa cells transfected with empty plasmids cultured under normoxia as a negative control. To determine whether SHARP1 altered the functional behavior of endothelial cells, HUVECs were placed on a basement membrane matrix to induce capillary-like tube formation in the presence of the CMs mentioned above. Tube formation was quantified by averaging the total length of tubes in three randomly chosen fields. Similarly, CM from SHARP1-overexpressing Ishikawa cells significantly inhibited hypoxia-stimulated tube formation and branching (Figure 3D). Thus SHARP1 was acting as an antiangiogenic molecule in culture under hypoxic conditions by inhibiting the proliferation, migration, and maturation of vascular endothelial cells. To confirm this effect in vivo, we performed a mouse angiogenic Matrigel plug assay. We mixed growth factor-reduced Matrigel with the CMs or corresponding Ishikawa cells mentioned above and inoculated these into mouse flanks. The plugs were removed 14 days after inoculation, and were stained with hematoxylin and eosin (H&E) or immunostained for CD34 (Figure S3). Cells were still viable within the plug, and no signs of surrounding tissues invasion were observed. CM or corresponding hypoxic Ishikawa cells transfected with empty plasmid significantly induced functional blood vessel formation in Matrigel, while CM or corresponding hypoxic Ishikawa cells that overexpressed SHARP1 reduced microvessel formation within Matrigel implants as determined by the MVD (Figure 3E and F). These combined results suggested that SHARP1 upregulation leads to an enormous decrease in proangiogenic events.

To further confirm that SHARP1 inhibits tumor angiogenesis, we performed MVD analysis on four cases from each grade of EEC (G1–G3) and four cases of non-EEC specimens (one case of clear cell EC and three cases of papillary serous EC), while normal endometrium tissues were used as a control. All tissues were selected randomly from biobank. The MVD was much lower in normal endometrium tissue sections than in EC specimens (Figure 3G). There was a significant inverse correlation between SHARP1 immunostaining levels and MVD in EC using Spearman’s correlation (*r* = –0.610, *P*<0.05; Figure 3H).

### SHARP1 Inhibits Cell Viability

**Figure 4:**
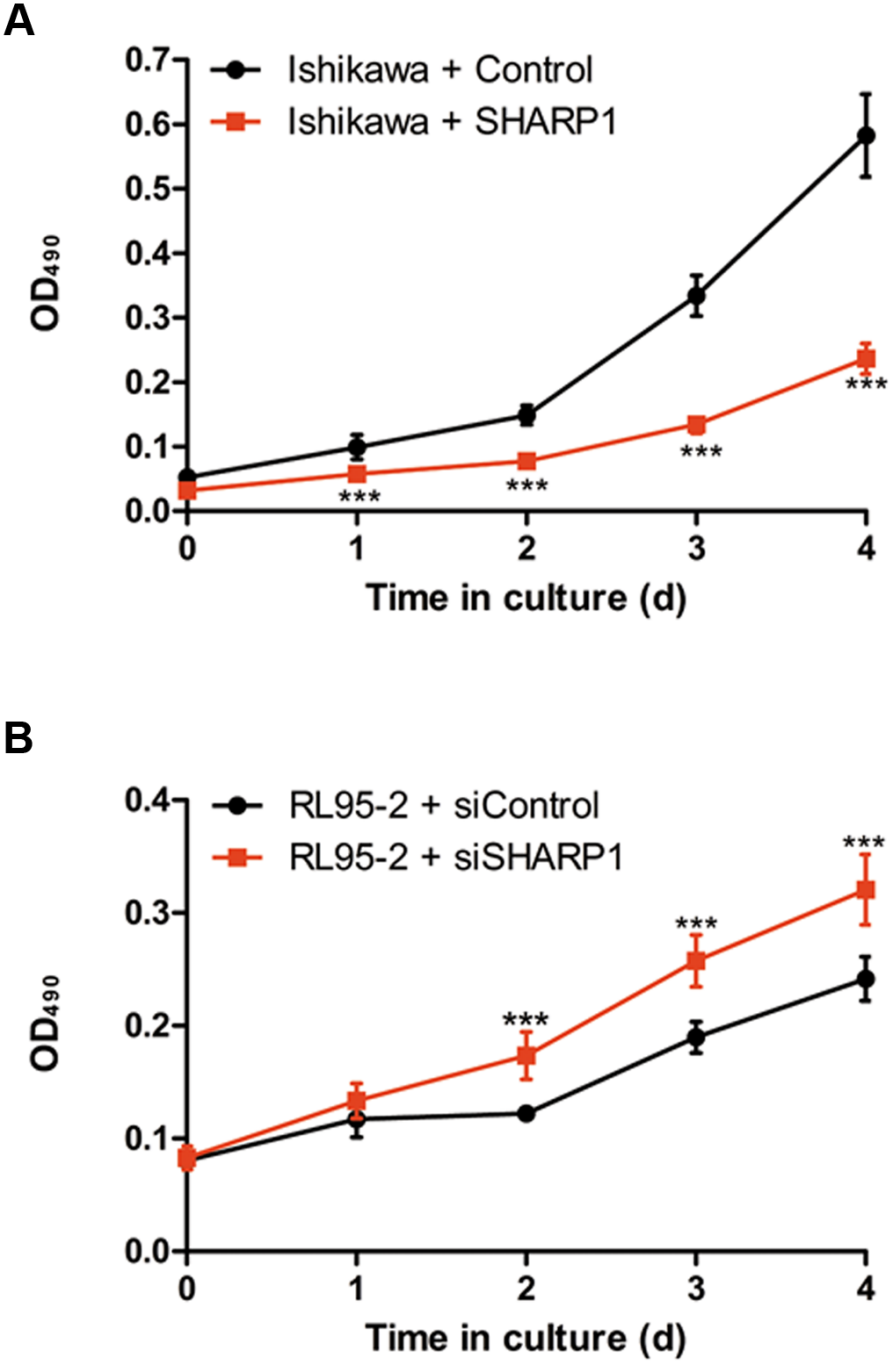
SHARP1 inhibits cell viability in EC cell lines. MTT assay was conducted at each time point to quantify cell viability for Ishikawa cells transfected with control or SHARP1 expression plasmid (A) and for RL95-2 cells transfected with control or SHARP1 siRNA (B). Data represent the mean ± SD from one representative experiment of three independent experiments, each performed in triplicate (***P<0.001).

We also tested the role of SHARP1 on cell viability in EC cell lines. Methyl thiazolyl tetrazolium (MTT) assays indicated that Ishikawa cell viability steadily decreased following transfection of the SHARP1 expression plasmid (Figure 4A), whereas RL95-2 cell viability steadily increased following siRNA transfection targeting SHARP1 (Figure 4 B).

### SHARP1 Suppresses Tumor Growth, Angiogenesis, and HIF-1α Expression in Tumor Xenografts

**Figure 5:**
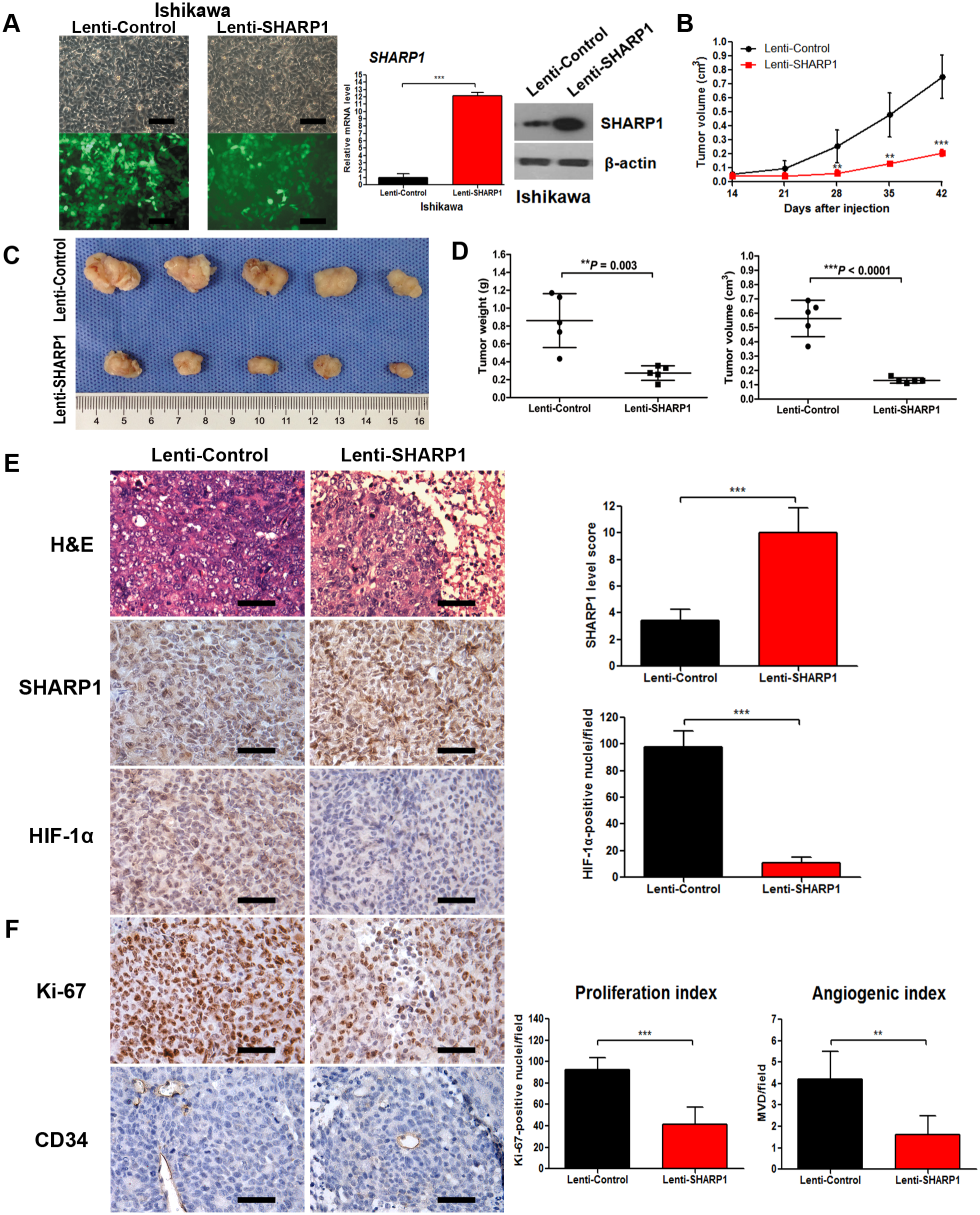
SHARP1 inhibits tumor growth, HIF-1α expression and angiogenesis in tumor xenografts. (A) Stable transfection of Ishikawa cells with empty lentiviral vectors or lentiviral vectors carrying human SHARP1 gene. Left panels showed morphology of Ishikawa cells under light microscope (upper) or fluorescence microscope (lower) in the same field (magnification: 200×; scale bar: 100 µm), and right panels showed transfection efficiency confirmed by qPCR and western blotting. Ishikawa cells transfected with Lenti-Control or Lenti-SHARP1 were injected subcutaneously into the flank of each mouse. (B) The mean tumor volume was measured by calipers on the indicated days. (C) Photographs of tumors excised 42 days after inoculation of stably transfected cells into nude mice. (D) Tumor weight (left) and volume (right) of each nude mouse at the end of 42 days. (E) Left: Representative microphotographs of H&E, SHARP1 and HIF-1α staining in nude mice tumor tissues (magnification: 400×; scale bar: 50 µm); Right: Statistical analysis of SHARP1 and HIF-1α staining in nude mice tumor tissues. (F) For evaluation of the proliferation index and angiogenic index, the Ki-67-stained nuclei and CD34-positive blood vessels in the hotspot areas were counted at 400× magnification. Representative photographs were taken at 400× magnification (scale bar, 50 µm). Data represent the mean ± SD of 5 grafts in each condition (**P<0.01, ***P<0.001).

To further investigate the function of SHARP1 in EC development, we constructed an Ishikawa cell line stably transfected with lentiviral vector expressing human SHARP1 for in vivo tumorigenicity assays (Figure 5A). Ishikawa cells transfected with the SHARP1 expression vector expressed more than 10-fold higher levels of *SHARP1* mRNA and increased levels of SHARP1 protein as compared with cells transfected with empty vector (Figure 5A). The cells were injected subcutaneously into the flank of each mouse. During a 6-week follow-up period, we observed a lower tumor growth rate in the Lenti-SHARP1 group (*P*<0.0001; Figure 5B). At 6 weeks, the sizes and weights of tumors were substantially smaller in the Lenti-SHARP1 group as compared with those of the Lenti-Control group (Figure 5C and D).

The tumor tissues were then embedded in paraffin and stained with hematoxylin and eosin (H&E) for histological examination. Upregulation of SHARP1 strongly decreased immunostaining of HIF-1α in grafted cells (Figure 5E). Ki67 staining was performed as a measure of cell proliferation. Ki67 expression occurred in the nuclei of tumor cells, and the Lenti-SHARP1 group had a lower proliferation index in the xenograft tumors (*P* = 0.0004; Figure 5F). In addition, the MVD was much lower in the Lenti-SHARP1 group than in the Lenti-Control group (*P* = 0.0062; Figure 5F).

## Discussion

High levels of VEGF expression are found in 56–66% of EC tumors [34]. VEGFA and MVD are independent predictors of a poor prognosis in patients with EEC [34]–[36]. Bevacizumab (a monoclonal antibody against VEGFA), the first antiangiogenic drug approved by the U.S. Food and Drug Administration, has been studied in recurrent EC and shows promising therapeutic effects [37], [38]. Our findings that VEGF secretion and VEGFA mRNA levels are correlated with different proangiogenic abilities of CMs in EC cell lines further support this point. Because antiangiogenic therapy is a promising option in EC treatment but accompanied with divergent therapeutic responses, we attempted to clarify the mechanism underlying angiogenesis regulation to seek a novel strategy and marker for antiangiogenic therapy.

The growth of EC is dependent on the development of neo-vessels. As tumor growth outstrips its vasculature, resulting in a hypoxic microenvironment, activation of the HIF-1α/VEGF axis plays a critical role in initiating tumor angiogenesis and progression [33]. Therefore, inhibiting hypoxia-induced angiogenesis is considered a potential strategy for attenuating tumorigenesis [39]. The physiological effect of HIF-1α can be regulated at different levels, including at the level of the transcriptional activity of the *HIF-1α* gene and the stability of its resulting mRNA, the accumulation of HIF-1α protein, and its downstream signaling pathways. In this study, hypoxic culture remarkably enhanced the level of HIF-1α protein, and the proangiogenic property of Ishikawa cells when cultured under hypoxic conditions confirmed this point. Recent studies suggest that various exogenous small-molecule inhibitors of HIFs may be useful as optional drugs for tumor therapy [39], [40]. To better understand and manipulate this pathophysiological process, we have focused on the effect of SHARP1, an endogenous and physiologic molecule, on the HIF-1α/VEGF axis.

SHARP1 is a bHLH transcription factor that is involved in different cellular processes including proliferation [41], apoptosis [21], [24], differentiation [42], and regulation of circadian rhythm [25], [43], [44]. Most previous studies acknowledge SHARP1 as a tumor suppressor, and our results support the idea that upregulation of SHARP1 impairs tumor angiogenesis and tumor growth in EC. In addition, SHARP1 is transcriptionally activated under hypoxia via hypoxia response elements in its promoter [25], [45]. However, unlike other hypoxia-induced genes, SHARP1 exerts repression effects on expression under hypoxia, implying its unique expression-regulatory mechanisms during tumorigenesis [25], [46]. In agreement with these findings, we observed an elevated SHARP1 level induced by hypoxia while knockdown of HIF-1α decreased SHARP1 expression under hypoxia. And for the first time we verified a negative correlation between the expression patterns of SHARP1 and HIF-1α in EC tissue specimens accompanied by a simultaneous inverse regulatory effect of SHARP1 on the HIF-1α/VEGF axis in EC cells. This suggests that a SHARP1-mediated feedback loop participates in the regulation of gene expression under hypoxia in EC cells.

It was reported that SHARP1 promoted degradation of HIF-1α regardless of oxygen concentration [23]. However, we could hardly detect the HIF-1α protein under normoxia in EC cells as HIF-1α degraded rapidly with the increase of oxygen concentration, and we also found that SHARP1 failed to suppress the downstream genes of HIF-1α under normoxia. Therefore, it was hardly to tell whether SHARP1 could regulate HIF-1α expression under normoxia in EC, which was partly different from the findings by Montagner et al. in breast cancer [23]. Nevertheless, we confirmed that SHARP1 inhibits *VEGFA*, *ANGPTL4*, and *CA9* under hypoxia, which play crucial roles in angiogenesis, metabolism, and pH regulation [47], in a HIF-1α-dependent manner. Additionally, a physical interaction between SHARP1 and HIF-1α was observed under hypoxia. Thus, this SHARP1/HIF-1α interaction may impede the ability of an EC cell to switch to an angiogenic phenotype under hypoxia, leading to suppression of tumor growth and progression in EC. However, the actual molecular mechanisms underlying the inverse regulation between and interaction of SHARP1 and HIF-1α are still unclear in EC and require further investigation. Moreover, angiogenesis is regulated by a balance of proangiogenic and angiostatic factors [11], [16]. Although VEGF is a central regulator in angiogenesis, SHARP1 may suppress other cytokines to impede angiogenesis, as a full proangiogenic signature was not analyzed [16].

Interestingly, Ishikawa cells, exposed to hypoxia for only 24 h, possessed an elevated pro-angiogenic property during a 14-day growth in Matrigel plug assays when compared to cells cultured under normoxia, implying a relatively long-lasting effect of hypoxia on cellular homeostasis. This suggests that occasional exposure to hypoxia induced either by treatments or endogenously present, as often observed in high-grade EC, could have profound long-term effects on the pro-angiogenic property and consequently tumor aggressiveness. The findings may also elucidate the divergent responses of endometrial cancers to antiangiogenic therapy. As antiangiogenic therapy accompanied with a risk of causing regional hypoxia within tumor [38], SHARP1 may be a valuable marker to patients who might benefit from antiangiogenic therapy. Further studies are required to clarify the mechanisms underneath in this respect.

In agreement with the role of SHARP1 in the suppression of angiogenesis, for the very first time, we also showed that decreased expression of SHARP1 is associated with blood vessel permeation in the myometrium, lymph node metastasis, myometrial invasion, and late-stage/high-grade tumors in EC patients.

To establish a cell line stably expressing SHARP1 in nude mice xenograft assay, the SHARP1 gene constructed in the lentiviral vectors was fused with green fluorescent protein (GFP). The fusion may disturb the fully expression of GFP, resulting in a disparity in immunofluorescence images. Nevertheless, qPCR and western blotting were performed to verify the transfection efficiency and significant elevated expression of SHARP1 mRNA and protein levels were observed. Therefore, we used these cells for following xenograft assay.

Identification of SHARP1 as a feedback regulator under hypoxia provides a new perspective for antiangiogenic therapy, as targeting of HIF-1α potentially provides an efficient means of inhibiting metastasis and overcoming therapeutic resistance [48]. However, EC progression consists of a series of rate-limiting steps in which HIF-1α may or may not be involved [49], [50]. Whether SHARP1 participates in other pathophysiological processes to contribute to malignant behaviors of EC is unknown and further studies are required.

In conclusion, we identified an important tumor-suppressive function for SHARP1 during EC progression for the first time, especially in the regulation of angiogenesis, and provided abundant clinical evidences. Moreover, we presented a mechanistic link between SHARP1 and HIF-1α, and provided clinical and functional evidence suggesting that this pathway is exploited during the progression of EC. As a factor that is transcriptionally activated under hypoxia but simultaneously exerts a suppressive role in hypoxia-promoted tumor progression, SHARP1 is considered a crucial member of a self-limiting system that regulates the cellular response to hypoxia. Taken together, these findings suggest that SHARP1 may be a valuable prognostic biomarker for EC progression. Our study has uncovered a novel pathway by which hypoxia-induced tumor pathogenesis could be manipulated and provides a new insight into future therapies targeting angiogenesis in this disease.

## Supporting Information

Figure S1 **Transfection efficiency in Ishikawa and RL95-2 cells.** qPCR (A) and western blot (B) analysis of SHARP1 overexpression efficiency in Ishikawa cells. qPCR (C) and western blot (D) analysis of SHARP1 knockdown efficiency in RL95-2 cells. For transient transfections, plasmid (1.6 µg/ml) and siRNA (40 pmol/ml) were used. For qPCR analyses, expression levels are relative to β-actin, and data were normalized to expression shown in the first column. Data represent the mean ± SD from one representative experiment of three independent experiments, each performed in triplicate (**P<0.01, ***P<0.001; NS, not significant).
(TIF)

Figure S2 **Knockdown of HIF-1α impacts expression of SHARP1 and its target genes.** (A) qPCR (left) and western blot (right) analysis of the effects of HIF-1α knockdown using siRNA in Ishikawa cells after hypoxic culture for 24 h. (B) qPCR analyses of *VEGFA*, *ANGPTL4*, and *CA9* in Ishikawa cells that had been transfected with control or HIF-1α siRNAs and incubated under hypoxia for 24 h. For transient transfections, siRNA (40 pmol/ml) were used 24 h before hypoxic culture. Data represent the mean ± SD from one representative experiment of three independent experiments, each performed in triplicate (*P<0.05, **P<0.01, ***P<0.001).
(TIF)

Figure S3 **H&E and CD34 staining in Matrigel plugs.** Representative microphotographs of H&E (A) and CD34 (B) staining in Matrigel plugs mixed with Ishikawa cells. (C) Representative microphotographs of CD34 staining in Matrigel plugs mixed with CMs. N: Ishikawa cells transfected with empty plasmid (1.6 µg/ml) under normoxia; H: Ishikawa cells transfected with empty plasmid (1.6 µg/ml) under hypoxia; H + S: Ishikawa cells transfected with SHARP1 full-length plasmid (1.6 µg/ml) under hypoxia; CM: conditioned medium. (magnification: 400×; scale bar: 50 µm).
(TIF)
