# Supplementary figures and images for: SHARP1 Suppresses Angiogenesis of Endometrial Cancer by Decreasing Hypoxia-Inducible Factor-1α Level
Source: PLoS One. 2014 Jun 11;9(6):e99907. doi: 10.1371/journal.pone.0099907 (PMC4053412; doi:10.1371/journal.pone.0099907)

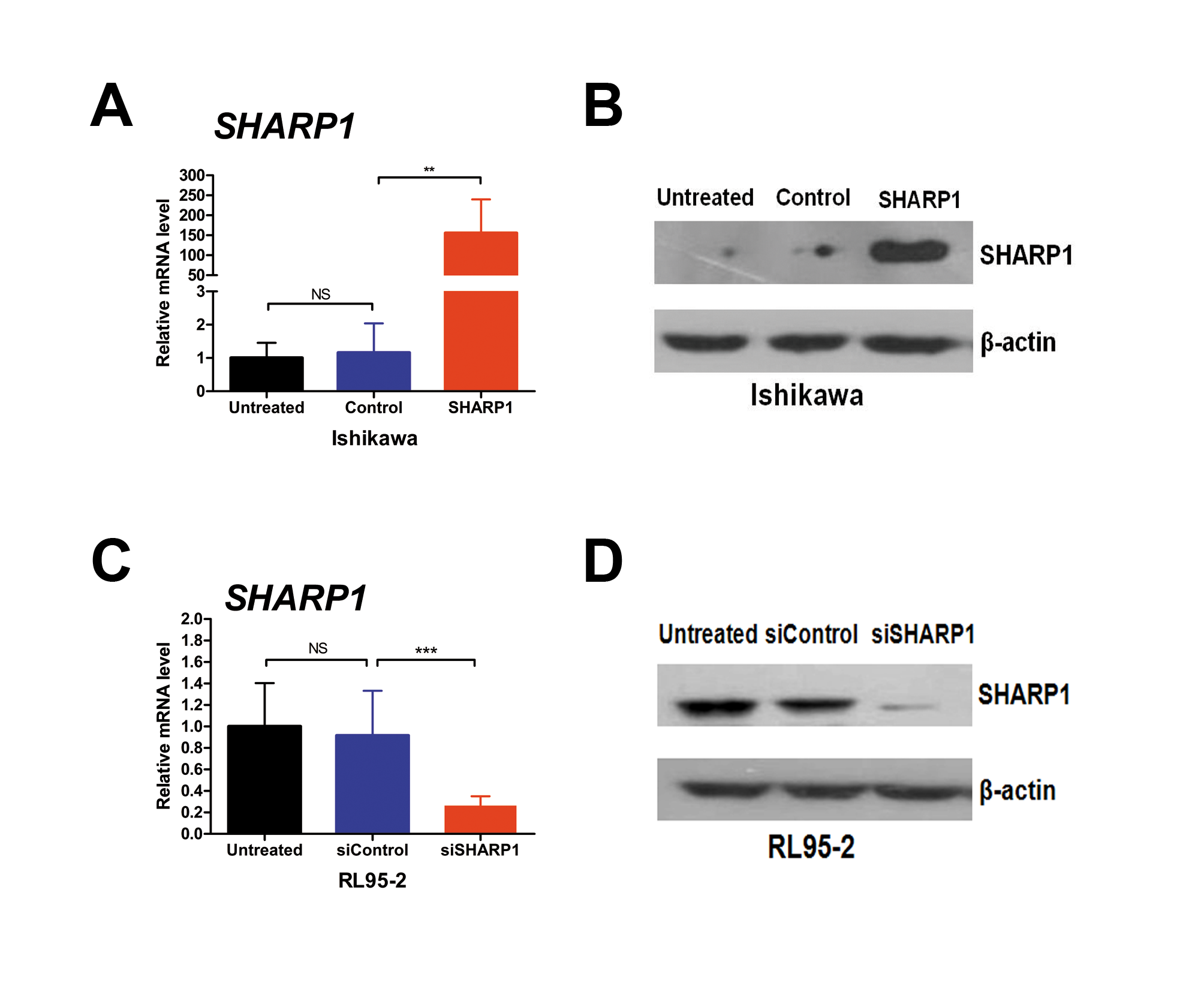

Supplement: Figure S1 — Transfection efficiency in Ishikawa and RL95-2 cells. qPCR (A) and western blot (B) analysis of SHARP1 overexpression efficiency in Ishikawa cells. qPCR (C) and western blot (D) analysis of SHARP1 knockdown efficiency in RL95-2 cells. For transient transfections, plasmid (1.6 µg/ml) and siRNA (40 pmol/ml) were used. For qPCR analyses, expression levels are relative to β-actin, and data were normalized to expression shown in the first column. Data represent the mean ± SD from one representative experiment of three independent experiments, each performed in triplicate (**P<0.01, ***P<0.001; NS, not significant). (TIF) [file pone.0099907.s001.tif]

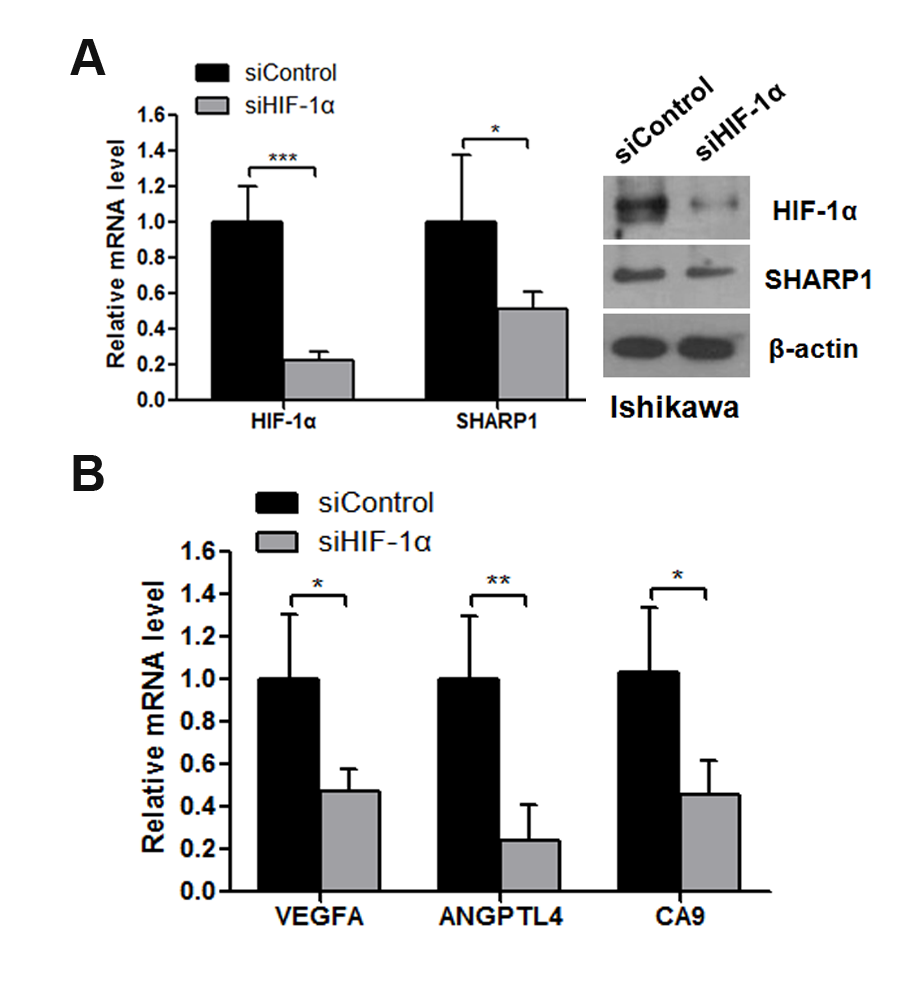

Supplement: Figure S2 — Knockdown of HIF-1α impacts expression of SHARP1 and its target genes. (A) qPCR (left) and western blot (right) analysis of the effects of HIF-1α knockdown using siRNA in Ishikawa cells after hypoxic culture for 24 h. (B) qPCR analyses of VEGFA, ANGPTL4, and CA9 in Ishikawa cells that had been transfected with control or HIF-1α siRNAs and incubated under hypoxia for 24 h. For transient transfections, siRNA (40 pmol/ml) were used 24 h before hypoxic culture. Data represent the mean ± SD from one representative experiment of three independent experiments, each performed in triplicate (*P<0.05, **P<0.01, ***P<0.001). (TIF) [file pone.0099907.s002.tif]

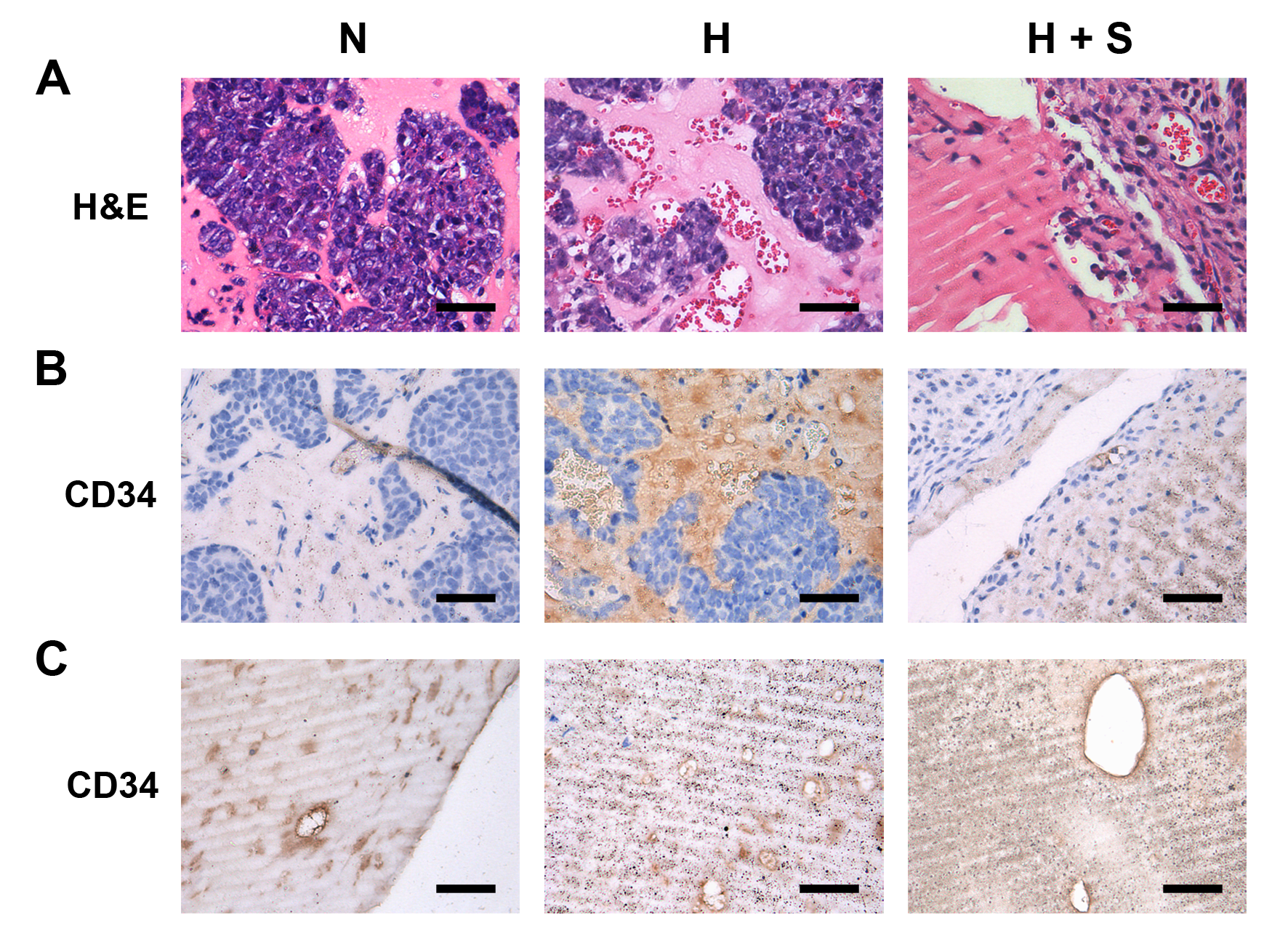

Supplement: Figure S3 — H&E and CD34 staining in Matrigel plugs. Representative microphotographs of H&E (A) and CD34 (B) staining in Matrigel plugs mixed with Ishikawa cells. (C) Representative microphotographs of CD34 staining in Matrigel plugs mixed with CMs. N: Ishikawa cells transfected with empty plasmid (1.6 µg/ml) under normoxia; H: Ishikawa cells transfected with empty plasmid (1.6 µg/ml) under hypoxia; H + S: Ishikawa cells transfected with SHARP1 full-length plasmid (1.6 µg/ml) under hypoxia; CM: conditioned medium. (magnification: 400×; scale bar: 50 µm). (TIF) [file pone.0099907.s003.tif]
